# An epidemiologic study of factors associated with nasal septum deviation by computed tomography scan: a cross sectional study

**DOI:** 10.1186/1472-6815-12-15

**Published:** 2012-12-17

**Authors:** Alireza Mohebbi, Aslan Ahmadi, Maryam Etemadi, Manouchehr Safdarian, Shadi Ghourchian

**Affiliations:** 1 Ear-Nose-Throat [ENT] and Head and Neck Surgery Research Center of Rasul-e-Akram Hospital, Tehran University of Medical Sciences, Sattarkhan street, Tehran, Iran; 2 Tehran University of Medical Sciences (TUMS), Students’ Scientific Research Center of TUMS, Tehran, Iran

**Keywords:** Sinusitis, Septal deviation, Concha bullosa

## Abstract

**Background:**

Sinusitis is an inflammation of the paranasal sinuses that can be caused by anatomic variations of the nasal cavity and paranasal sinuses. In this study we aimed to find the relationship between sinusitis and septal deviation (SeD) and concha bullosa.

**Methods:**

Two trained resident of ENT evaluated sinus CT scans of 463 cases presenting with nasal obstruction or chronic sinusitis symptoms from April 2011 to December 2011. CT scans were checked for the presence of conchae bullosa and the degree of septal deviation. The severity of sinusitis was evaluated according to the Lund Mackay criteria. The frequency of patients with different degrees of SeD and different grades of chronic sinusitis were studied.

**Results:**

Of 463 cases, 47% had septal deviation. Concha bullosa was seen in 16.8% of the patients in the left side and 27.6% of them in the right side. There was no significant relationship between the presence of concha bullosa and the severity of sinusitis. Also the P value of analytical tests between the severity of sinusitis, osteomeatal involvement and the degree of septal deviation was not significant. Analysis of the relationship between the presence of SeD (either to right or left) and the severity of sinusitis in different sinuses revealed no significant P value.

**Conclusions:**

By this study, the relationship between concha bullosa in osteomeatal complex and the severity of sinusitis was not cleared. No relationship was found between the severity of sinusitis, osteomeatal involvement and the degree of septal deviation. Also SeD (either to right or left) was not found to be associated with the severity of sinusitis in different sinuses.

## Background

Sinusitis usually caused by any disorders contributed to the blockage of the osteomeatal unit (OMU) such as infections, allergies and disorders of mucociliary transport. Also, anatomic variations of the nasal cavities and paranasal sinuses are other factors that may cause blockage [1].

Anatomical variants of the sinonasal cavities are very common and about 15 major variants have been described [2]. Variations causes narrowing or obstruction of the osteomeatal channels, thereby hamper the normal airflow and mucociliary clearance of the sinuses. Some studies contend that an anatomic variation is a factor in development of chronic sinusitis ;depend on its size, location or the amount of mucosal contact caused by the variation [3].

Nasal septum deviation is a common disorder that presents in up to 62% of the population, and its role in the pathogenesis of chronic sinusitis remains uncertain [4].

A concha bullosa is a pneumatized cavity within a turbinate in the nose [5]. The incidence rate of this disorder has varied from 14 to 53% in different studies. The relationship between the presence of concha bullosa and sinusitis has not been clarified yet [6] . While some studies suggest that septal deviation(SeD) or the presence of concha bullosa may interfere with proper airflow, potentially predisposing to sinus diseases, other studies have produced contradictory findings [7,8].

Since the relationship between the site of chronic sinusitis and the degree of nasal septum deviation has not been established by previous studies, this study was designed with a large sample size to investigate any associations between the presence of septal deviation and its degree with the severity of sinusitis and also the relationship between the presence of concha bullosa and the severity of sinusitis.

The result can help therapeutic strategies. Surgeries (septoplasty) associated with SeD can decrease the rate of chronic sinusitis and so its complications if there is a correlation between these two diseases.

## Methods

### Ethical approval

This study was a cross sectional study that was approved by ear-nose-throat (ENT) and Head and Neck surgery research center of Rasoul-e-Akram hospital of Tehran university of medical sciences.

Written informed consent was obtained from the patient for publication of this research and any accompanying images.

### Design

From April 2011 to December 2011, all patients presented with nasal obstruction or chronic sinusitis symptoms to ENT clinic were explained about the aim and design of the study. To confirm the diagnosis of chronic sinusitis, all patients should underwent sinus CT scan (PNC CT) as a gold standard. From those patients who agreed to be entered in the investigation, were selected to be evaluated for the severity of sinusitis according to the Lund Mackay staging system [9].

Also the CT scans were checked for the presence of conchae bullosa and the degree of SeD. If the patient had a history of nasal trauma within the last year or had a major chronic disease, he/she was excluded from the study. Also heavy smokers (>10 pack/year) were not included the study.

**Figure 1:**
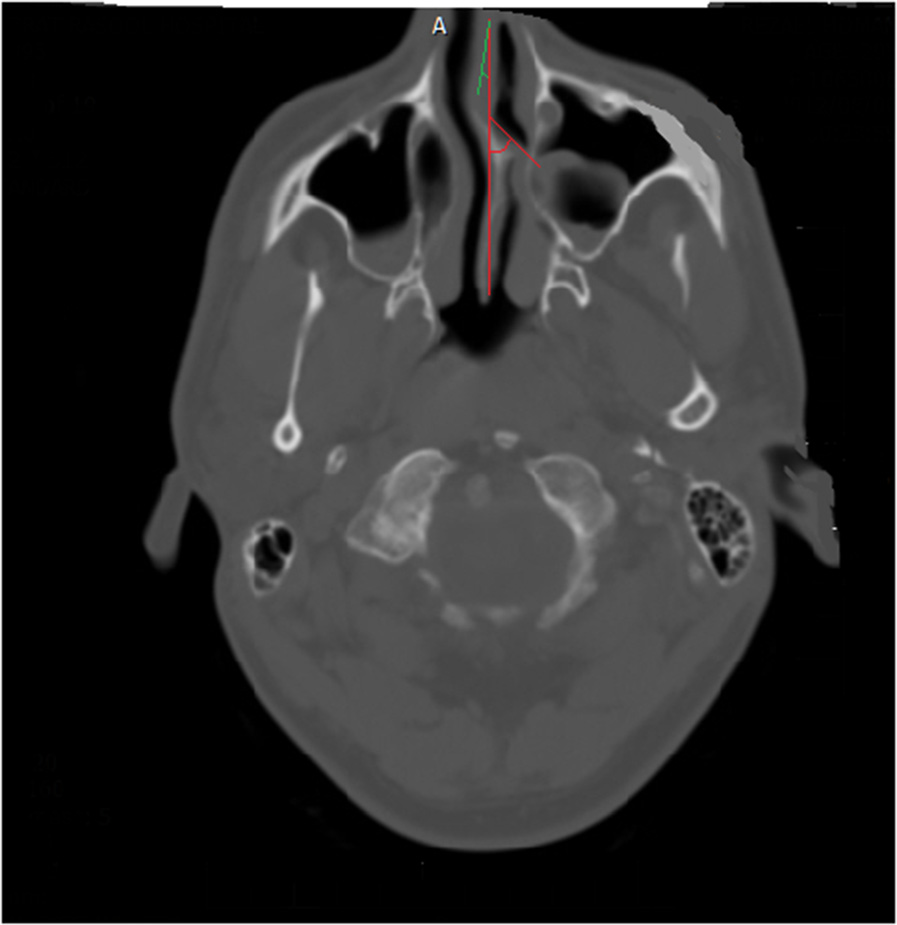
**The severity of maxillary sinus was considered as 1 if there was an incomplete involvement.** If there was an S shape deviation in septum, the more deviated angle was considered for analysis. In this picture, the septum is deviate first to left (green color) and then to right (red color), but because the deviation to right was much more than the other side, the first deviation to left was ignored.

To define the severity of sinusitis in different sinuses, we used Lund-Mackay scoring system. If the sinus was completely involved the score was considered 2, if there was not a complete involvement the score was considered 1 (Figure 1), and if the sinus was completely intact the score was considered 0.

According to the degree of SeD, the patients were categorized to 4 groups; the first group (normal group): 0 < SeD ≤ 5 (degree of the center), the second group (mild): 5 < SeD ≤ 10, the third group (moderate): 10 < SeD ≤ 20 and the forth group (severe): SeD > 20. If there was an S shape deviation, the more deviated angle to right or left was considered as the SeD for analysis (Figure 1). The patients whose CT scans did not reveal any chronic inflammations of sinuses were excluded. At the end of the period of the study, 463 patients were entered for analyzing.

### Statistical analysis

The data were entered to the SPSS software, version 18 (PASW). To describe the qualitative variables, the percentage was used as the descriptive index and to describe the quantitative variables, mean and standard deviation (SD) were used.

To find the difference between the degree of SeD in each group of patients with different severity of sinusitis, chi square test was used. Also to understand the relationship between the severity of sinusitis and also the degree of SeD in patients with conchae bullosa with the group of patients without concha bullosa, chi square test was used. The P value less than 0.05 was considered as significant. To clarify the relationship between SeD to right/ left and the site of involvement, the patients with the opposite site of deviation were omitted and the severity of the disease in normal population (without SeD) was compared with the severity of the disease in patients with right or left involvement.

## Results

At the end of the study, 463 cases were analyzed. 47% of them had septal deviation. The frequency of different grades of chronic sinusitis and the involved site are shown in Table 1.

**Table 1:** **Frequency of different grades of chronic sinusitis** (%)

	**Mild**	**Moderate**	**Severe**
R.M	72.8	12.3	14.9
L.M	73.2	11	15.8
R.A.E	76.5	8.4	15.1
L.A.E	77.3	8	14.7
R.P.E	81.4	3.7	14.9
L.P.E	81.9	4.3	13.8
R.F	87.5	3	9.5
L.F	87.9	2.8	9.3
R.S	87	3.7	9.3
L.S	86.4	2.4	11.2
R.OMC	73.9	10.8	15.3
L.OMC	75.8	8.2	15.8

*R*: right, *L*: left, *A*: anterior, *P*: posterior, *M*: maxillary sinus, *E*: ethmoid sinus, *F*: frontal sinus, *S*: sphenoid sinus, *OMC*: osteomeatal complex.

In our study, concha bullosa was seen in 16.8% of the patients in the left side and 27.6% of them in the right side.

In our study, 47% of patients had septal deviation. The frequency of patients with different degrees of SeD is shown in Table 2.

**Table 2:** **Frequency of patients with different degrees of septal deviation** (%)

	**Normal(0 < SeD ≤ 5)**	**Mild(5 < SeD ≤ 10)**	**Moderate(10 < SeD ≤ 20)**	**severe(SeD > 20)**
Deviated to left	53%	7.6	7.4	4.3
Deviated to right		9.3	12.8	5.6

The patients were evaluated for the bilateral involvements (Table 3).

**Table 3:** **The frequency of different sites of involvement** (**moderate or severe involvement**) (%)

	**Right involvement**	**Left involvement**	**Bilateral involvement**
Conchae bullosa	27.6	16.8	8.4
Maxillary sinus	27.2	27	18.4
Frontal sinus	12.5	12.1	11.2
Anterior ethmoid sinus	23.5	22.7	19.7
Posterior ethmoid sinus	18.6	18.1	16.4
Sphenoid sinus	13	13.8	12.3
Osteomeatal complex	26.1	24.2	17.5

The results of chi square test between the presence of concha bullosa and the severity of sinusitis is shown in Table 4.

**Table 4:** **The P value of the analysis between the presence of concha bullosa and the severity of sinusitis**, **osteomeatal involvement**

	**R**.**M**	**L**.**M**	**R**.**A**.**E**	**L**.**A**.**E**	**R**.**P**.**E**	**L**.**P**.**E**	**R**.**F**	**L**.**F**	**R**.**S**	**L**.**S**	**R**.**OMC**	**L**.**OMC**
Right conchae bullosa	0.07	0.9	0.03	0.06	0.003	0.002	0.09	0.1	0.057	0.09	0.1	0.1
Left conchae bullosa	0.0001	0.7	0.004	0.09	0.008	0.02	0.03	0.1	0.01	0.055	0.002	0.4

*R*: right, *L*: left, *A*: anterior, *P*: posterior, *M*: maxillary sinus, *E*: ethmoid sinus, *F*: frontal sinus, *S*: sphenoid sinus, *OMC*: osteomeatal complex.

The results of chi square test between the severity of sinusitis and the degree of SeD is shown in Table 5.

**Table 5:** **The P value of the analysis between the severity of sinusitis**, **osteomeatal involvement and the degree of septal deviation**

	**R**.**M**	**L**.**M**	**R**.**A**.**E**	**L**.**A**.**E**	**R**.**P**.**E**	**L**.**P**.**E**	**R**.**F**	**L**.**F**	**R**.**S**	**L**.**S**	**R**.**OMC**	**L**.**OMC**
Deviation to right	0.08	0.6	0.3	0.3	0.2	0.6	0.8	0.3	0.1	0.1	0.2	0.3
Deviation to left	0.3	0.8	0.5	0.6	0.8	0.9	0.6	0.6	0.7	0.1	0.3	0.8

*R*: right, *L*: left, *A*: anterior, *P*: posterior, *M*: maxillary sinus, *E*: ethmoid sinus, *F*: frontal sinus, *S*: sphenoid sinus, *OMC*: osteomeatal complex.

The relationship between the presence of SeD (either to right or left) and the severity of sinusitis was calculated (Table 6).

**Table 6:** **The relationship between the presence of SeD** (**either to right or left**) **and the severity of sinusitis** (**P value**)

	**R**.**M**	**L**.**M**	**R**.**A**.**E**	**L**.**A**.**E**	**R**.**P**.**E**	**L**.**P**.**E**	**R**.**F**	**L**.**F**	**R**.**S**	**L**.**S**	**R**.**OMC**	**L**.**OMC**
Septal deviation to right	0.4	0.2	0.7	0.2	0.8	0.9	0.7	0.7	0.2	0.7	0.6	0.03
Septal deviation to left	0.8	0.3	0.6	0.4	0.4	0.9	0.5	0.7	0.7	0.1	0.4	0.3

## Discussion

In lots of previous studies, the increased degree of SeD was in association with the increased prevalence of rhinosinusitis. A review article of five articles in 2010 revealed a significant relationship between the presence of SeD and rhinosinusitis (P value of chi square analysis = 0.0004, odds ratio = 1.47). By reviewing the literatures, in lots of studies that examined the laterality of rhinosinusitis and septal deviation, inflammation was found bilaterally. Also, it seems that previous studies were insufficiently powered to detect an association between rhinosinusitis and the degree of septal deviation. In the other hand, the severity of sinusitis has not been sufficiently investigated [10].

In a study conducted between 2003 and 2005 to evaluate the recurrence of the symptoms of rhinosinusitis in 130 patients, 58 patients were involved by both nasal septal deviation and concha bullosa. In that study, no statistical differences were found between the presence of sinusitis and SeD. Also they did not find any relationship between the SeD and frontal recess or osteomeatal involvement. Of 130 patients, 44.6% were involved by both nasal septal deviation and concha bullosa. In that study, the prevalence of sinusitis in patients with severe SeD was significantly more than the patients with mild to moderate deviation [11].

In another study, PNC CT scan of 44 patients with rhinosinusitis were evaluated to understand the relationship between the presence of concha bullosa, SeD and sinusitis. They found a significant relationship between unilateral concha bullosa and SeD. In the other hand, they did not find any significant relationships between the presence of unilateral and bilateral concha bullosa and sinusitis. Also the presence of bilateral concha bullosa was not significantly associated with SeD (p > 0.05) [12].

In another study, CT scans of orbit in 89 cases with no history of sinusitis were studied to investigate the incidence of the presence of septal deviation, concha bullosa, paradoxical middle turbinates and the correlations. The results showed that despite radiological findings, there were no significant relationships between these abnormalities and an increased incidence of sinusitis [13]. In another similar study, no statistical relationship was found between SeD and sinusitis. They revealed that middle turbinate changes and SeD are not associated with chronic sinusitis [14].

A study in the last decade showed no significant difference between the presence of SeD in patients with chronic rhinosinusitis and the control group. Also they did not find any significant relationship between the severity of SeD and OMC involvement with the severity of sinusitis [15].

In a study in 2008, 883 CT scans taken at Creighton University School of Dentistry reviewed for the presence of concha bullosa, nasal SeD, and maxillary sinusitis. Concha bullosa was seen in 67.5% that 49.3% of them had maxillary sinusitis. Also19.4% of patients had a deviated septum, and 50.0% of them had mucosal thickening consistent with maxillary sinusitis. There was no significant relationship between the presence of concha bullosa or nasal septal deviation and maxillary sinusitis [16].

In another study, 100 PNC CT scans were compared with 82 orbital CT scans. Concha bullosa was associated with anterior ethmoid disease (p less than 0.04). Septal deviation was associated with osteomeatal complex disease (p less than 0.01) and with anterior (p less than 0.04) and posterior (p less than 0.04) ethmoid disease [17].

Lots of previous studies investigated the association between septal deviation and sinusitis that revealed some significant results. Comparing our findings with the previous studies which assessed the relationship between septal deviation / its degree and the severity of sinusitis and also the relationship between concha bullosa and the severity of sinusitis, the results were the same.

## Conclusion

Since there was no coordination between our findings, the exact conclusion of association between the presence of concha bullosa and the severity of sinusitis or osteomeatal involvement has not been raised. To define the relationships between the presence of concha bullosa and the severity of sinusitis meta-analysis based on other previous findings seems to be needed.

By our study, it is concluded that there is no association between the severity of sinusitis, osteomeatal involvement and the degree of septal deviation which is in contrast with some previous studies but confirm the others (Table 5).

Moreover, we did not find any associations between the presence of SeD (either to right or left) and the severity of sinusitis in different sinuses (Table 6). The presence of septal deviation and its degree were not associated with the severity of sinusitis in our study. Also the presence of concha bullosa was not seemed to have a clear relationship with the severity of sinusitis.

## Abbreviations

SSRC: Scientific Students’ Research Center of Tehran University of Medical Sciences; SeD: Septal deviation; OMU: Osteomeatal unit; ENT: Ear-nose-throat; PNC CT: Sinus CT scan.

## Competing interests

The authors declare that they have no competing interests.

### Financial competing interests

In the past five years we have not received reimbursements, fees, funding, or salary from an organization that may have any financial gains from the publication of this manuscript, either in now or in future.

We do not hold any stocks or shares in an organization that may in any way gain or lose financially from the publication of this manuscript, either now or in the future.

We do neither hold nor currently applying for any patents relating to the content of the manuscript.

We have not received reimbursements, fees, funding, or salary from an organization that holds or has applied for patents relating to the content of the manuscript.

We have not any financial competing interests. Non-financial competing interests.

There are not any non-financial competing interests (political, personal, religious, academic, ideological, intellectual, commercial or any other).

## Authors’ contributions

AM: study concept and design; acquisition, approved the final manuscript. AA: study concept and design, coordination for the acquisition of data, read and approved the final manuscript. ME: study concept and design approved the final manuscript the final manuscript. MS: Acquisition, interpretation of data, writing the manuscript. ShGh: Acquisition, Designing the study, analysis and interpretation of data, writing the manuscript, read and approved the final manuscript.

## Pre-publication history

The pre-publication history for this paper can be accessed here:

http://www.biomedcentral.com/1472-6815/12/15/prepub
